# Supplementary material for: Study on the Role of Salicylic Acid in Watermelon-Resistant Fusarium Wilt under Different Growth Conditions
Source: Plants (Basel). 2022 Jan 22;11(3):293. doi: 10.3390/plants11030293 (PMC8839013; doi:10.3390/plants11030293)
Supplement: Supplementary file 1 [file plants-11-00293-s001.zip › supplementary file plants-1531695/Figure S1. The standard curve of SA content. 20220124.pdf]

Figure S1. The standard curve of SA content.

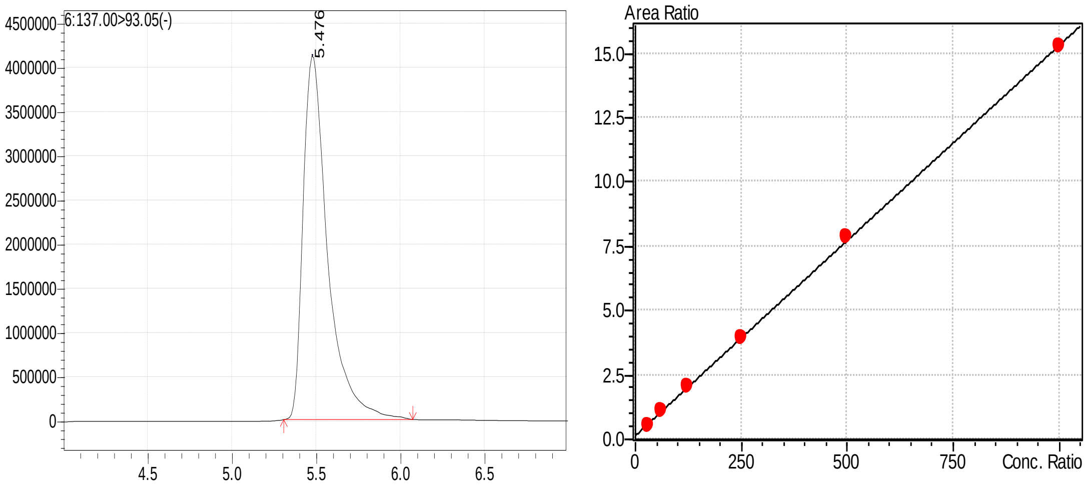


Note: Three biological replicates per samples were analyzed.
