# Supplementary material for: Study on the Role of Salicylic Acid in Watermelon-Resistant Fusarium Wilt under Different Growth Conditions
Source: Plants (Basel). 2022 Jan 22;11(3):293. doi: 10.3390/plants11030293 (PMC8839013; doi:10.3390/plants11030293)
Supplement: Supplementary file 1 [file plants-11-00293-s001.zip › supplementary file plants-1531695/Table S1. The disease incidences of Fusarium wilt under watermelon monocropping system. 20220121.pdf]

Table S1. The disease incidences of Fusarium wilt under watermelon monocropping system.

| Year | Disease incidence | S.E. |
|---|---|---|
| 2016 | 93 | 0.27 |
| 2017 | 80 | 0.23 |
| 2018 | 98.68 | 0.66 |
| 2019 | 99.58 | 0.49 |

Note: Three biological replicates per samples were analyzed. Data were expressed as mean±SE (n=3).
