# Supplementary material for: Study on the Role of Salicylic Acid in Watermelon-Resistant Fusarium Wilt under Different Growth Conditions
Source: Plants (Basel). 2022 Jan 22;11(3):293. doi: 10.3390/plants11030293 (PMC8839013; doi:10.3390/plants11030293)
Supplement: Supplementary file 1 [file plants-11-00293-s001.zip › supplementary file plants-1531695/Table S2. Specific primer sequences of 20 candidate genes used for qRT-PCR. 202201021.pdf]

Table S2. Specific primer sequences of 20 candidate genes used for qRT-PCR.

Gene ID	Gene name	Forward primer (5'-3')	Reverse primer (5'-3')
Cla008727	*ClPAL1*	ATGAAACCCAACACTTGCAAT	ACTCCCCCACCATTTTCTT
Cla011180	*ClPAL2*	AAGGCTGCCAACGCTCTCAG	ATCGCTTCGACGAGCAACGG
Cla018297	*ClPAL3*	GCCGGAACAATATCAACCATG	CATCCGCTTCACTTCTTCC
Cla018298	*ClPAL4*	TGCGCCATTACTACTCATCC	GCGCTTCACCTCATTGAG
Cla018299	*ClPAL5*	TGACTTGAGGCACCTAGAAG	ATCTTGAAGGATGAAGTGTGC
Cla018300	*ClPAL6*	TGGCACCAACTCAATTCCAG	CCTCATCGAGATGGCTTCC
Cla018301	*ClPAL7*	TGCCCAATTGGAACAATGTG	TATACTCTTCCACCATCCTCTT
Cla018302	*ClPAL8*	TGGAGACCATCTGCAAGAG	GCGCTTCACCTCATCAAG
Cla018303	*ClPAL9*	CCCTAGTCAAGACCATTTGCAA	GCTTGACAAGAGGCCTCCGA
Cla012779	*ClPAL10*	ATGGCCCAAAAGGTTTGTGCT	ATACTCTTCCACCATCCGC
Cla012780	*ClPAL11*	GGCTTCCAAAAATAATGATTC	TCCTCCAAGCTTCACCAAA
Cla013761	*ClPAL12*	ATCCGGAAAATGGCAACCAT	CCTCCAAGTCGCACAATAG
Cla019128	*ClICS*	ACGGAGAGTCTGAGGAGAG	GCACTTGAAGCCGAATAATC
Cla002899	*NPR1*	TCTTGGCGGCTAGGAGTTTG	GTCGTGATGGTGGTGGTGAT
Cla019154	*NPR*	TCTTGATGCTCCTCTCCCGA	CCAACGATCGCATTCCGTTC
Cla003370	*WRKY*	AGAAGAAGATAAGAAAAGGG	CGTAGGTTGTTAATACCACTC
Cla022362	*WRKY3*	CAGGCTCCAGCCATTCTTGC	TTATCGTCGCTACAGCAACC
Cla002084	*WRKY6*	GACTGTCGGCGTATCATCAT	CGCCGCATCCATCTCTGTCT
Cla005515	*WRKY29*	GCGACGTTGGATCCTGCTTT	GACGACGACGACACTGAAGA
Cla010867	*WRKY72*	GCTGTTTCGCGACGTTCCGA	TTGTCGTCGTTCCCGCCAT
Cla007792	*ClACT*	CCATGTATGTTGCCATCCAG	GGATAGCATGGGGTAGAGCA

Note: The watermelon *ClACT* gene was used as reference gene.
